# Case Report: A rare presentation of pulmonary tuberculosis with extensive ground-glass opacities in an immunocompetent patient: lessons from metagenomic next-generation sequencing

**DOI:** 10.3389/fmed.2026.1696371

**Published:** 2026-03-11

**Authors:** Juanjuan Mao, Qian Jin, Dan Ye, Yide Yang

**Affiliations:** Department of Infectious Diseases, Taizhou Municipal Hospital (Taizhou University Affiliated Municipal Hospital), School of Medicine, Taizhou University, Taizhou, China

**Keywords:** diagnosis, ground-glass opacity, metagenomic next-generation sequencing, therapy, tuberculosis

## Abstract

Pulmonary tuberculosis (PTB) is typically diagnosed through sputum smear microscopy and culture. However, diagnosis is challenging in patients with atypical radiological features and negative conventional tests. Ground-glass opacities (GGOs) are common but non-specific computed tomography (CT) findings and are rarely observed in immunocompetent PTB patients. We report the first case of an immunocompetent 53-year-old female presenting with extensive bilateral GGOs without classic clinical symptoms. Conventional microbiological cultures, acid-fast staining, and serological assays were all negative. Metagenomic next-generation sequencing (mNGS) of bronchoalveolar lavage fluid identified *Mycobacterium tuberculosis*, further supported by a positive T-spot TB assay. Standard anti-tuberculosis therapy led to complete resolution of GGOs over nine months, confirmed by follow-up CT imaging. This case underscores the diagnostic challenge of atypical PTB presenting with non-classical CT manifestations in an immunocompetent host. It highlights the decisive role of mNGS as a complementary tool in cases where conventional methods fail, enabling timely diagnosis, precise treatment, and improved patient outcomes.

## 1 Introduction

Tuberculosis (TB) remains one of the most severe global health threats, with an estimated 10.8 million cases and 1.25 million deaths reported worldwide in 2024 (1). Despite decades of public health interventions, early and accurate diagnosis of pulmonary tuberculosis (PTB) continues to be a formidable challenge. Conventional diagnostic methods, such as sputum smear microscopy, mycobacterial culture, and nucleic acid amplification tests, are widely available but demonstrate suboptimal sensitivity in patients with atypical or paucibacillary disease. These limitations contribute to diagnostic delays, ongoing transmission, and worse clinical outcomes.

Radiological examination, particularly chest computed tomography (CT) (2), plays a central role in evaluating suspected PTB. The classical CT features of PTB include cavitation, centrilobular nodules, tree-in-bud appearance, and miliary nodules in disseminated cases. However, atypical radiological manifestations are increasingly recognized, especially in elderly, immunocompromised, or subclinical cases. Ground-glass opacities (GGOs) (3–8) are among the most frequent but non-specific CT findings, commonly associated with viral pneumonia, fungal pneumonia, interstitial lung disease, hypersensitivity pneumonitis or alveolar hemorrhage. In the context of PTB, GGOs are rare, and when present, they usually accompany reversed halo signs (9–11) or disseminated disease in patients with impaired immunity. Reports of extensive bilateral GGOs as the predominant radiological manifestation in immunocompetent PTB patients are virtually absent in the literature.

Such atypical presentations pose substantial diagnostic dilemmas. On the one hand, patients may lack classical respiratory symptoms such as cough, sputum production, afternoon hectic fever, night sweats or hemoptysis; on the other hand, routine microbiological assays frequently return negative results due to the low bacillary burden. In this setting, clinicians are confronted with non-specific radiological patterns and inconclusive laboratory findings, delaying treatment initiation and increasing the risk of disease progression or misdiagnosis as viral or fungal infection.

Metagenomic next-generation sequencing (mNGS) has emerged as a powerful diagnostic tool to overcome these limitations (12–15). By enabling unbiased sequencing of microbial nucleic acids, mNGS can detect almost all known pathogens in a single assay, independent of prior assumptions or culture requirements. Recent studies (16–20) have demonstrated its utility in identifying *Mycobacterium tuberculosis* in sputum-negative patients and in complex mixed infections. However, its application in cases with atypical imaging presentations and immunocompetent hosts has rarely been documented.

In the present manuscript, we first peer-reviewed and fully documented report of an immunocompetent patient with PTB manifesting as extensive bilateral GGOs, in whom mNGS provided the decisive evidence for diagnosis. By sharing this case, we aim not only to contribute to the understanding of atypical imaging PTB presentations but also to provide practical insights into the integration of mNGS into diagnostic workflows for challenging infectious diseases. This report demonstrates that mNGS enables early identification and timely diagnosis of pulmonary tuberculosis with atypical radiological features, laying a foundation for the early recognition of such atypical cases. Early diagnosis facilitates subsequent precise treatment, reduces the transmission of *Mycobacterium tuberculosis*, contributes to the control of the tuberculosis epidemic, and lowers the medical costs for patients.

## 2 Case presentation

A 53-year-old female medical worker, with no known history of immunodeficiency, chronic pulmonary disease, or systemic comorbidities, presented with low-grade afternoon fever and menstrual irregularity of one week’s duration. She denied cough, phlegm, chest tightness, chest pain, night sweats, or hemoptysis. Importantly, she had undergone pulmonary nodule surgery 1.5 years earlier, with postoperative pathology revealing only intrapulmonary dust deposition, suggesting no prior active infectious disease. She was otherwise healthy, with no history of diabetes, malignancy, long-term corticosteroid use, or known TB exposure.

**FIGURE 1:**
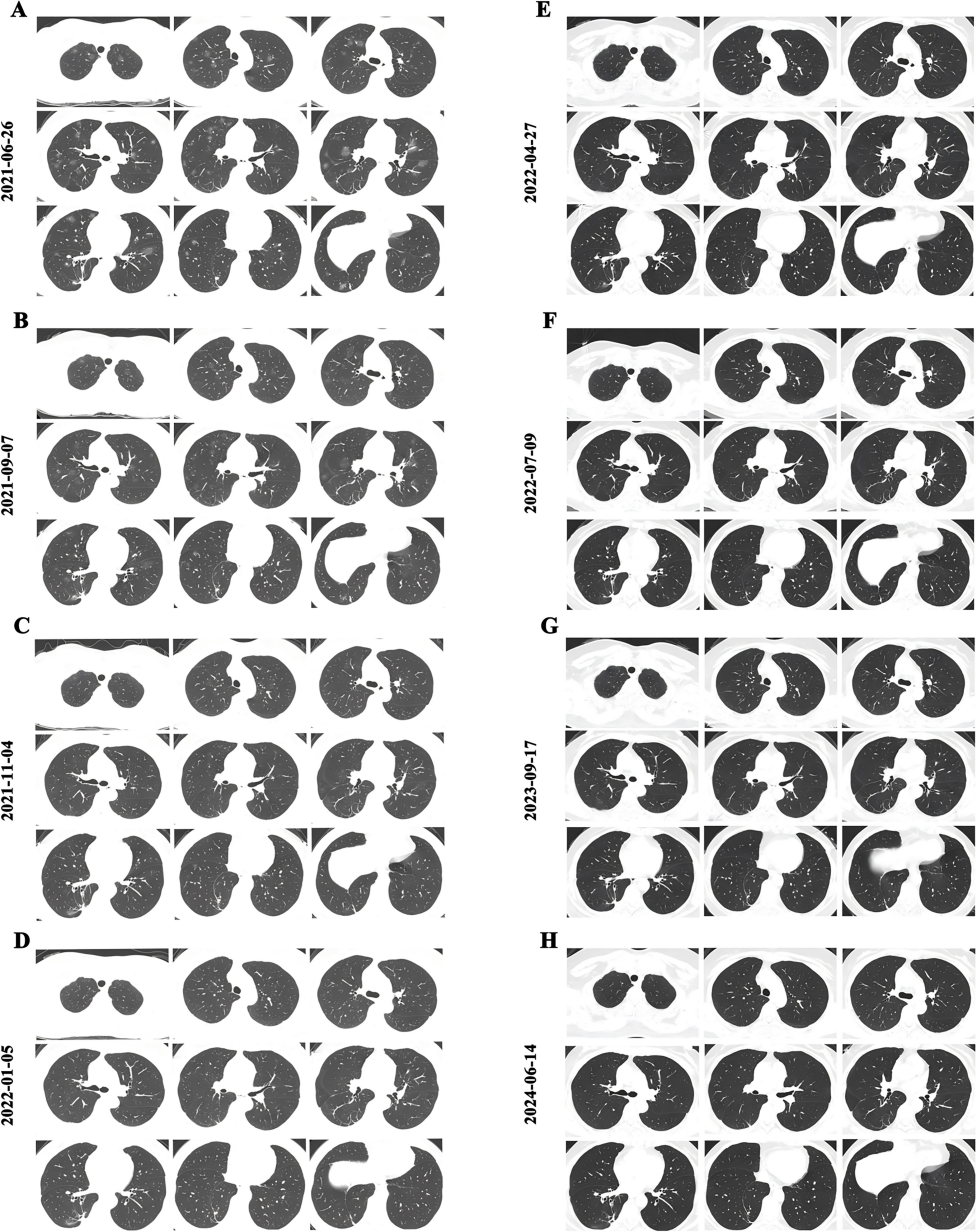
Chest CT images before and after anti-tuberculosis treatment (2HRZE/7HR regimen). **(A)** Baseline CT showing extensive bilateral ground-glass opacities (GGOs); **(B)** After 2 months of treatment, multiple GGOs had slightly decreased in size and density compared with baseline; **(C)** After approximately 4 months, GGOs further decreased in extent and density compared with **(B)**; **(D)** After 6 months, CT demonstrated a marked reduction of GGOs; **(E)** After 9 months of treatment, at the end of therapy, CT revealed complete resolution of GGOs with no new lesions; **(F–H)** Follow-up CT scans at approximately 2, 15, and 25 months after treatment discontinuation showed stable findings, with no recurrence of GGOs or new pulmonary lesions.

She had a physical examination at a local hospital on 26 June 2021, and her chest CT showed multiple GGO with clear boundaries in both lungs, along with a few patchy shadows in the lower lobe of the right lung (Figure 1A). Given the imaging appearance, fungal infection was initially suspected. As the patient exhibited no respiratory symptoms, the discrepancy between radiological abnormalities and mild systemic manifestations raised clinical uncertainty, prompting referral to a tertiary care hospital for further evaluation.

On admission (1 July 2021), physical examination was unremarkable, and initial laboratory tests—including serum β-d-Glucan test (G test), cryptococcal capsular antigen test (CrAg test), glactomannan test (GM), and severe acute respiratory syndrome coronavirus 2 (SARS-CoV-2) were negative. Complete blood counts, erythrocyte sedimentation rate (ESR), total immunoglobulin E (IgE), autoantibodies (ANA, ANCA), and immunoglobulin panels were within normal limits, excluding systemic autoimmune or allergic disease.

Bronchoscopy was subsequently performed. Microscopic examination of bronchoalveolar lavage fluid (BALF) revealed no abnormal cytology. Multiple acid-fast stains, routine bacterial cultures, and mycobacterial cultures were all negative. Despite the extent of radiological lesions, the absence of identifiable pathogens led to diagnostic uncertainty. At this juncture, metagenomic next-generation sequencing (mNGS) of BALF was ordered to broaden the search for infectious etiologies.

**FIGURE 2:**
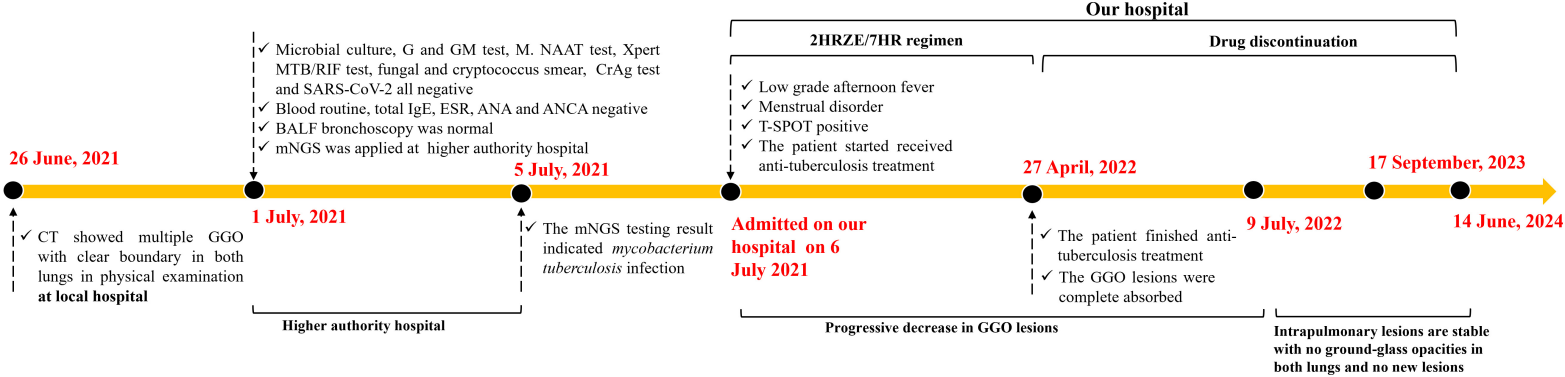
Clinical timeline of the reported case.

The patient was discharged temporarily on 3 July 2021, while awaiting sequencing results. On July 5, mNGS revealed multiple microbial reads, including *Staphylococcus aureus* (4 reads), *Enterococcus faecalis* (20 reads), and *Prevotella melaninogenica* (13 reads), but these were interpreted as colonizers given the absence of corresponding clinical symptoms. Crucially, seven reads corresponding to *Mycobacterium tuberculosis* were detected, with a genomic coverage of 0.0236% (Table 1 and Figure 2). Although the read count was low, the finding was consistent with the clinical suspicion of tuberculosis. A T-spot. The TB test was subsequently performed and yielded positive results, supporting the diagnosis.

**TABLE 1:** Pathogens detected by mNGS.

Type	Genus	Species	Coverage (%)	Depth	Number of reads
G^+^	*Staphylococcus*	*Staphylococcus aureus*	0.0071	1	4
G^+^	*Enterococcus*	*Enterococcus faecalis*	0.0443	1	20
G^+^	*Mycobacterium tuberculosis* complex	*Mycobacterium tuberculosis*	0.0236	1	7
G^–^	*Prevotella*	*Prevotella melaninogenica*	0.0344	1	13

On 12 July 2021, the patient commenced a standard anti-tuberculosis regimen consisting of isoniazid, rifampicin, ethambutol, and pyrazinamide (2HRZE/7HR). Serial CT monitoring was conducted to assess radiological response. After two months of treatment, the GGOs showed partial reduction in size and density (Figure 1B). At four months, further absorption was evident (Figure 1C), and by six months, a marked resolution of most lesions was observed (Figure 1D). Following nine months of treatment, GGOs had completely resolved with no new lesions (Figure 1E).

**FIGURE 3:**
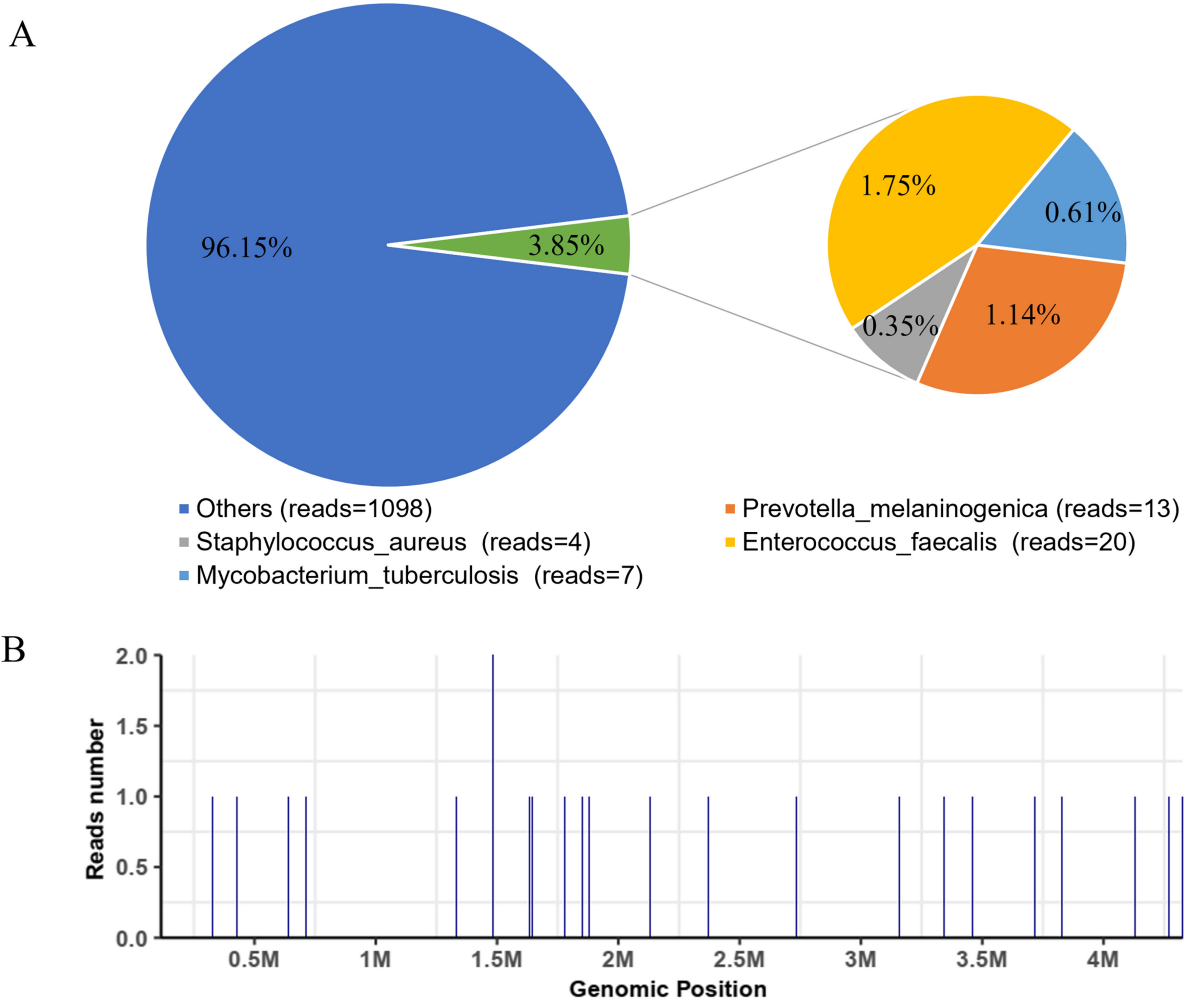
Results of metagenomic next-generation sequencing (mNGS) of the patient’s bronchoalveolar lavage fluid (BALF). **(A)** Coverage plot of pathogen genomic DNA; **(B)** Detection of *Mycobacterium tuberculosis* reads by mNGS.

Long-term follow-up with dynamic CT scans at approximately 2, 15, and 25 months post-therapy confirmed stable remission, with no recurrence or emergence of new pulmonary abnormalities (Figures 1F–H). Notably, throughout the treatment course, repeated acid-fast staining and mycobacterial cultures remained negative, underscoring the pivotal role of mNGS in establishing the diagnosis. The timeline of the patient’s clinical course described above is presented in Figure 3.

## 3 Discussion

Ground-glass opacities are common radiological findings but lack specificity, being associated with a broad spectrum of infectious, inflammatory, and neoplastic processes (3–8). In clinical practice, GGOs are most frequently linked to viral pneumonias, interstitial lung diseases, alveolar hemorrhage, fungal pneumonia, interstitial lung disease, hypersensitivity pneumonitis, or pulmonary edema. In contrast, pulmonary tuberculosis (PTB) is rarely reported to present primarily as extensive GGOs, particularly in immunocompetent individuals. Most documented cases of PTB with GGOs occur in patients with impaired immunity, such as those with HIV infection, hematological malignancies, or immunosuppressive therapy, where granuloma formation is compromised, and diffuse alveolar involvement ensues (9, 11).

In classical PTB, chest CT commonly demonstrates cavitation, centrilobular nodules, and tree-in-bud opacities reflecting endobronchial spread. In disseminated TB, a miliary pattern with innumerable tiny nodules is characteristic. The reversed halo sign (RHS), consisting of a central GGO surrounded by a ring of consolidation, has also been described in some TB cases, although it remains a rare presentation (9–11). However, extensive and bilateral GGOs without cavitation or miliary nodules, as observed in our patient, are exceedingly uncommon. To our knowledge, this is the first reported case of active PTB manifesting in this manner in an immunocompetent host.

The pathophysiological mechanisms underlying GGOs in PTB remain incompletely understood. GGOs generally reflect partial airspace filling, interstitial thickening, or a combination of both. In TB, this may occur due to early alveolar exudation before granuloma maturation, hematogenous dissemination with microvascular injury, or atypical immune responses leading to diffuse alveolar damage. It is conceivable that in our patient, localized immune responses generated an atypical radiological signature despite preserved systemic immunity. Further histopathological correlation in future cases is needed to clarify the biological basis of these imaging patterns.

Another challenge highlighted by this case is the limitation of conventional diagnostic methods in atypical PTB. Despite extensive radiological abnormalities, multiple sputum and BALF acid-fast smears, mycobacterial cultures, and fungal serologies remained negative. This reflects the well-known problem of low sensitivity of smear and culture in paucibacillary or atypical presentations. Such scenarios can delay diagnosis, lead to inappropriate treatment (e.g., empirical antifungal or antiviral therapy), worsens patient outcomes and leads to the further dissemination of *Mycobacterium tuberculosis*, thereby triggering the occurrence of tuberculosis outbreaks (2, 15).

The World Health Organization (WHO) advocates a stratified diagnostic strategy and specifies that the rapid pathway of “screening → rapid molecular diagnosis → treatment” should be adopted in high TB-burden settings. As a high TB-burden country, China regards rapid molecular diagnosis such as Xpert MTB or mNGS as critically important. Several recent studies have confirmed that mNGS has higher sensitivity and specificity than conventional culture for TB diagnosis, particularly in sputum-negative cases, and can reduce the time to diagnosis significantly (15, 17). Metagenomic next-generation sequencing provided a breakthrough in this case. Although only a small number of *Mycobacterium tuberculosis* reads were detected, the unbiased sequencing approach enabled identification of the pathogen that all conventional methods failed to capture. The finding was further corroborated by a positive T-spot. TB assay and, most importantly, the patient’s dramatic clinical and radiological response to anti-tuberculosis therapy. Our case extends this evidence by demonstrating that mNGS can also resolve diagnostic uncertainty in patients with atypical imaging and immunocompetent status.

This study explores the importance of mNGS in the timely and early diagnosis of atypical PTB and establishes an inherent link with the WHO End TB Strategy (1), which emphasizes that early diagnosis and early treatment are key to tuberculosis prevention and control. To the best of our knowledge, this study is the first to highlight that GGO in the lung should raise suspicion for PTB, providing an important reminder for the early identification of such patients.

Furthermore, conventional diagnostic methods show low positive rates in these patients. We recommend timely mNGS testing to achieve early definitive diagnosis. mNGS-informed precision treatment to improve cure rates and reduce mortality. This will reduce the further spread of *Mycobacterium tuberculosis*, lower the transmission of pulmonary tuberculosis, facilitate tuberculosis prevention and control, and contribute to the achievement of the WHO’s 2035 goal of ending the tuberculosis epidemic.

Precise treatment of PTB can reduce the use of empirical medication and optimize the allocation of medical resources by lowering the overall diagnostic and therapeutic costs for patients.

From a clinical perspective, this case provides several important lessons. First, PTB should remain in the differential diagnosis of patients presenting with unexplained bilateral GGOs, even in the absence of classical symptoms or immunodeficiency. Second, repeated negative conventional microbiological results should not preclude PTB, especially when clinical suspicion remains high. Third, mNGS can serve as a powerful complementary tool, particularly in diagnostically challenging cases, by providing broad-spectrum and unbiased pathogen detection.

Looking forward, we propose a health research agenda for the future. The incorporation of mNGS into diagnostic workflows may help overcome current bottlenecks in TB diagnosis. While its cost and availability remain barriers to routine use, the development of rapid, affordable, and point-of-care (POC) mNGS technologies suitable for resource-constrained settings and targeted application in atypical or diagnostically ambiguous cases could be highly cost-effective by preventing delayed or missed diagnoses. Furthermore, systematic studies integrating mNGS findings with radiological and immunological data may provide new insights into the diverse phenotypic spectrum of PTB and the host-pathogen interactions that underlie atypical imaging features such as GGOs.

This case report has several limitations. Given the availability of clinical data, patient perspective was not included in this case report. The pathological basis of the CT manifestations in this immunocompetent PTB patient remains unclear. Multiple GGOs with well-defined margins were observed, a pattern that can also be seen in patients with a reversed halo sign or hematogenously disseminated PTB; however, neither of these subtypes was diagnosed in this case. PTB accompanied by GGOs in immunocompetent individuals is rare, and additional cases are needed to clarify the underlying etiology. Furthermore, the high cost of mNGS compared with conventional diagnostic methods limited its use as an initial test in this patient. Nevertheless, mNGS offers clear advantages, including high sensitivity, broad pathogen coverage, and rapid turnaround time. By enabling precise pathogen identification in infections of unknown origin, mNGS can guide accurate therapy and help reduce the risk of disease progression.

## 4 Conclusion

In conclusion, we present the first reported case of pulmonary tuberculosis in an immunocompetent patient manifesting with extensive bilateral ground-glass opacities. This atypical presentation, combined with repeatedly negative conventional tests, underscores the diagnostic challenges of such cases. Metagenomic next-generation sequencing proved decisive by enabling timely identification of *Mycobacterium tuberculosis* and guiding effective treatment. Our findings highlight the value of mNGS as a complementary diagnostic tool in atypical PTB, supporting more precise and timely patient care. This approach not only reduces the diagnostic and therapeutic costs for patients but also decreases the dissemination of *Mycobacterium tuberculosis*, contributing to the control of the tuberculosis epidemic.

## Data Availability

The datasets presented in this study can be found in online repositories. The names of the repository/repositories and accession number(s) can be found in this article/supplementary material.
